# Gas Chromatography, GC/Mass Analysis and Bioactivity of Essential Oil from Aerial Parts of *Ferulago trifida*: Antimicrobial, Antioxidant, AChE Inhibitory, General Toxicity, MTT Assay and Larvicidal Activities

**Published:** 2017-09-08

**Authors:** Saeed Tavakoli, Hassan Vatandoost, Reza Zeidabadinezhad, Reza Hajiaghaee, Abbas Hadjiakhoondi, Mohammad Reza Abai, Narguess Yassa

**Affiliations:** 1 Department of Pharmacognosy, Faculty of Pharmacy and Medicinal Plant Research Center, Tehran University of Medical Sciences, Tehran, Iran; 2 Medicinal Plants Research Center, Institute of Medicinal Plants, ACECR, Karaj, Iran; 3 Department of Medical Entomology and Vector Control, School of Public Health, Tehran University of Medical Sciences, Tehran, Iran; 4 Department of Environmental Chemical Pollutants and Pesticides, Institute for Environmental Research, Tehran University of Medical Sciences, Tehran, Iran

**Keywords:** *Ferulago trifida*, Larvicidal property, Antioxidant activity, Antimicrobial effect, MTT assay

## Abstract

**Background:**

We aimed to investigate different biological properties of aerial parts essential oil of *Ferulago trifida* Boiss and larvicidal activity of its volatile oils from all parts of plant.

**Methods:**

Essential oil was prepared by steam distillation and analyzed by Gas chromatography and GC/Mass. Antioxidant, antimicrobial, cytotoxic effects and AChE inhibitory of the oil were investigated using DPPH, disk diffusion method, MTT assay and Ellman methods. Larvicidal activity of *F. trifida* essential oil against malaria vector *Anopheles stephensi* was carried out according to the method described by WHO.

**Results:**

In GC and GC/MS analysis, 58 compounds were identified in the aerial parts essential oil, of which E-verbenol (9.66%), isobutyl acetate (25.73%) and E-β-caryophyllene (8.68%) were main compounds. The oil showed (IC_50_= 111.2μg/ml) in DPPH and IC_50_= 21.5 mg/ml in the investigation of AChE inhibitory. Furthermore, the oil demonstrated toxicity with (LD_50_= 1.1μg/ml) in brine shrimp lethality test and with (IC_50_= 22.0, 25.0 and 42.55 μg/ml) on three cancerous cell lines (MCF-7, A-549 and HT-29) respectively. LC_50_ of stem, root, aerial parts, fruits, and flowers essential oils against larvae of *An. stephensi* were equal with 10.46, 22.27, 20.50, 31.93 and 79.87ppm respectively. In antimicrobial activities, essential oil was effective on all specimens except *Escherichia coli*, *Aspergillus niger* and *Candida albicans.*

**Conclusion:**

The essential oil showed moderate antioxidant activity, strong antimicrobial properties and good toxic effect in brine shrimp test and MTT assay on three cancerous cell lines.

## Introduction

The use of medicinal plants to treatment of diseases and improve the health has always been attractive for men. In addition to traditional medicinal purposes, the plants are of most interest to researchers because of isolation and identification of bioactive compounds as the lead compound in the development and production of new drugs with efficacy and safety.

*Ferulago trifida* Boiss, from Apiaceae family is an Iranian indigenous plant and no study has been done for identification of its compounds in Iran, therefore phytochemical study on its extract, essential oil and some investigation e.g. antimicrobial and antioxidant effects, inhibition of the acetyl-cholinesterase and cytotoxic properties are indispensable on this valuable plant.

*Ferulago trifida* is an endemic plant in Iran that grows in Qazvin Province near the village of Qvanin Alamut region. This genus consists of seven species in Iran, most of them are endemic to Iran or have spread in regions of Anatolia, Syria, Lebanon, and Iraq, they are valuable pasture plants (1, 2). In certain parts of Iran, some species of this genus are traditionally added to dairy products, especially in the oil made of animal fats for a pleasant taste and presentation of corruption (3).

Different species of this plant in some parts of Turkey were used as a sedative, tonic, gastrointestinal pain, cure hemorrhoids etc. There is a report about the use of the essential oil of various species of this genus for preparation of cosmetic products (3). In recent years, researchers have studies on immune modulatory and cytotoxic effects of some medicinal plants that *F. angulata* (Schlecht) Boiss, is one of them (4, 5). During the studies on essential oil of *F. carduchorum*, antibacterial and antifungal effects on *Staphylococcus aureus* and *Candida albicans* as well as strong antioxidant effects have been observed (6).

Chemical analysis and antimicrobial activity of essential oil of *F. Bernardii* shown volatile oil of the plant contains 2, 4, 5-trimethylbenzaldehyde, caryophyllene oxide, spathulenol, cis-chrysanthenyl acetate, and α-pinene. Anti-microbial activity of the plant compared to fluconazole and gentamicin represents weak effects against *C. albicans*, *Aspergillus niger*, *Bacillus subtilis*, *S. aurous* and *Escherichia coli.* The volatile oil did not show any activity on *Pseudomonas aeruginosa* (7).

In several studies on some species of genus *Ferulago* different coumarins have been identified, from the two species *F. capillaris* and *F. brachyloba*, four new coumarins: (+)-3′-hydroxyprantschimgin, (−)-3′-senecioyloxymarmesin, senecioyloxymarmesin and (+)-senecioylprangol, have been detected (8). Acetylcholinesterase inhibitory effects of three isolated coumarins (umbelliprenin, coladonin, coladin), phenolic compounds, polyacetylene and daucane from *F. campestris* were studied; all of these compounds showed inhibitory effects on AChE (9).

In a study from Turkey on chloroform extract of *F. aucheri*, two coumarins: osthenol, prantschimgin and two flavonoids: isorhamnetin-3-glucoside, 6-hydroxyapigenin-6-methyl ether and two new aromatic compounds: quinolmono acetate, 1-acetylhydroquinone-4-galactoside were identified (10).

In this project, we studied larvicidal properties against *Anopheles stephensi* of *F. trifida* essential oils from its different parts (flower, leaf, aerial parts, root, stem, and fruits), and identified the essential oil components of aerial parts. In addition, we investigated antimicrobial, antioxidant, cytotoxic effects on Brine shrimp and three tumor cell lines, and acetylcholine esterase inhibitory effect of the volatile oil from aerial parts of *F. trifida*.

## Materials and Methods

### Plant material

The aerial parts of *F. trifida* were collected in July 2014 from Qazvin Province near the village of Ovan in Alamut region, Iran. The plant was identified by Dr V Mozaffarian (Research Institute of Forest and Rangelands, Tehran, Iran) and a voucher specimen was deposited in the herbarium of Faculty of Pharmacy, Tehran University of Medical Sciences, Tehran, Iran (No.THE-6562).

### Preparation of essential oils

The collected plant was dried in shade and powdered, 100g of powdered plant was subjected to hydrodistillation with a Clevenger type apparatus for 4h. The yellow color essential oil was dried over anhydrous sodium sulfate and was kept in refrigerator until analyses.

### Gas chromatography and GC/Mass spectroscopy

Essential oil of the aerial parts of *F. trifida* was analyzed on an HP-6890 gas chromatograph with an HP-5MS column (30×0.25mm id, 0.25 μm film thickness), equipped with HP-5973 mass detector (Ionization energy: 70 eV) under the following conditions, temperature program: 60 °C (0–3min), 60 °C to 250 °C at the rate of 3 °C/min (3–65 min), injector temperature: 220 °C, detector temperature: 290 °C, injection volume: 1.0μl, split ratio: 1:90, carrier gas: helium (Flow rate: 1ml min^−1^). The Kovats retention indices (KI) were calculated for all identified compounds using a homologous series of *n*-alkanes (C_8_–C_24_) injected under the same chromatographic conditions described for samples. The components of the oils were identified by comparison of their mass spectra and retention indices with Wiley library and those published in the literature (11). For quantitative analysis, essential oil was also injected to HP-6890 gas chromatograph with an HP-5MS column fitted with FID detector in conditions equal to GC/MS analysis (Table 1).

**Table 1.** Essential oil components of *Ferulago trifida* aerial parts by Gas Chromatography Mass spectroscopy

**No**	**Compounds**	**%**	**KI**
1	Hexanal	0.47	807
2	2*E*-hexenal	2.55	860
3	Nonane	0.20	900
4	Heptanal	0.34	909
5	*A*-pinene	0.42	935
6	Thuja-2,4(10)-diene	0.21	958
7	1-octen-3-ol	1.26	986
8	Myrcene	0.41	992
9	Mesitylene	1.16	1000
10	N-octanal	0.13	1011
11	*P*-cymene	0.22	1030
12	Limonene	0.44	1034
13	*(Z)-β*-ocimene	3.33	1040
14	*(E)-β*-ocimene	0.56	1050
15	*Γ*-terpinene	0.39	1063
16	Terpinolene	0.14	1090
17	Linalool	0.69	1106
18	N-nonanal	1.43	1113
19	*Allo*-ocimene	0.33	1133
20	*Cis*-verbenol	2.55	1151
21	*Trans*-verbenol	9.66	1156
22	*P*-mentha-1,5-dien-8-ol	3.22	1183
23	Terpinen-4-ol	0.36	1190
24	*P*-cymen-8-ol	0.40	1199
25	A-terpineol	0.30	1206
26	N-decanal	1.43	1214
27	Verbenone	0.22	1221
28	Geraniol	1.59	1258
29	*Cis*-verbenyl acetate	0.29	1288
30	Isobornyl acetate	25.73	1292
31	Thymol	2.35	1303
32	Carvacrol	1.30	1311
33	Undecanal	0.37	1316
34	*A*-copaene	0.52	1381
35	Geranyl acetate	0.31	1384
36	*B*-bourbonene	0.93	1389
37	*Z*-jasmone	0.38	1406
38	*(Z)*-caryophyllene	0.12	1411
39	*(E)*-caryophyllene	8.68	1427
40	*B*-copaene	0.23	1437
41	Neryl acetone	0.39	1456
42	*A*-humulene	0.81	1464
43	*Γ*-muurolene	0.43	1482
44	Germacrene D	2.18	1489
45	*B*-himachalene	0.64	1505
46	*B*-bisabolene	0.38	1514
47	*Δ*-cadinene	0.35	1526
48	*E*-nerolidol	0.21	1567
49	Geranylbutanoate	0.33	1575
50	Spathulenol	0.51	1590
51	Caryophyllene oxide	2.47	1595
52	Tetradecanal	0.14	1621
53	1-epi-cubenol	0.14	1640
54	Caryophylla-4(14),8(15)-diene-5-*α*-ol	0.29	1649
55	Caryophylla-4(14),8(15)-diene-5-*β*-ol	0.60	1652
56	*A*-muurolol	0.27	1656
57	*A*-cadinol	0.32	1670
58	14-hydroxy-9-*epi-(E)*-caryophyllene	0.33	1686
	**Total Identified**	**84.41**	

### Antibacterial activity

#### Bacterial strains

Antimicrobial activity of the essential oils was individually assessed against a set of seven bacterial strains, Gram-positive bacteria *S. aureus* (ATCC 29737), *S. epidermidis* (ATCC 12228) and *Bacillus subtilis* (ATCC 6633), Gram-negative bacterial *Pseudomonas aeruginosa* (ATCC 27853), *E. coli* (ATCC 10536), *Klebsiella pneumonia* (ATCC 10031), *Shigella dysenteriae* (PTCC 1188), *Salmonella paratyphi*-A (ATCC 5702) and *Proteus vulgaris* (PTCC 1182), as well as tree fungi including two mold, *Aspergillus brasiliensis* (ATCC 1015) and *A. niger* (ATCC 16404) and one yeast, *C. albicans* (ATCC 10231), provided from Iranian Research Organization for Science and Technology (IROST).

#### Disk diffusion assay

Disc diffusion method was applied for the evaluation of antimicrobial activity of essential oil (12). The essential oil was filtered through 0.45μm Millipore filters for sterilization. 100μl of suspension containing 10^8^ CFU/ml of bacteria, 10^4^ spore/ml of mold and 10^6^ CFU/ml of yeast were spread on the nutrient agar (NA), potato dextrose agar (PD) and sabouraud dextrose (SD) agar mediums, respectively. The impregnated discs (6mm in diameter) with 10μl of the essential oil were placed on the inoculated agar. The diameters of inhibition zones (IZ) (mm) were measured following incubation of all plates at 37 °C (bacteria) and at 30 °C (fungi) for 24h, gentamicin (10μg/disc) and rifampin (5 μg/disc) were used as positive controls for bacteria and nystatin (100I.U./disc) for fungi. Each assay was repeated twice and diameters of inhibition zones were represented as mean (Table 2).

**Table 2.** Antibacterial and antifungal activities of the essential oil of *Ferulago trifida* aerial parts

**Microorganisms**	**Essential oil of aerial parts**		**Antibiotics**					

			**Rif^a^**		**Gen^b^**		**Nys^c^**	

	**IZ^d^**	**MIC^e^**	**IZ**	**MIC**	**IZ**	**MIC**	**IZ**	**MIC**
*S. paratyphi-A*	20	500	-	-	21	500	NA^f^	NA
*S. aureus*	23	500	10	250	21	500	NA	NA
*S. epidermidis*	20	125	8	250	18	500	NA	NA
*E. coli*	-	-	11	500	20	500	NA	NA
*K. pneumoniae*	11	250	7	250	22	250	NA	NA
*B. subtilis*	16	125	13	15	21	500	NA	NA
*P. vulgaris*	11	250	10	125	23	500	NA	NA
*S. dysenteriae*	23	125	40	250	35	500	NA	NA
*C. albicans*	-	-	NA	NA	NA	NA	33	125
*A. brasiliensis*	12	500	NA	NA	NA	NA	23	500
*A. niger*	-	-	NA	NA	NA	NA	27	31

a dash (−) indicates no antimicrobial activity.

a Rifampin (5μg/disc),

b Gentamicin (10μg/disc),

c Nystatin (100 I.U. /disc),

d Inhibition zone in diameter (mm) around the impregnated discs including diameter of the disc (6mm) [weak activity (<10 mm), moderate activity (10–15mm), strong activity (15–20mm), very strong activity (20< mm)],

e Minimal inhibition concentrations as μg/ml,

f Not applicable.

#### Micro-well dilution assay

Essential oil was subjected to micro-well dilution assay in order to determination of minimal inhibition concentration (MIC) values, for microbial strains found susceptible in disc diffusion assay (13). The suspensions of microbial strains were prepared from their 12h broth cultures at 0.5Mc Farland standard turbidity. The serial two-fold dilutions of essential oil sample were made in a concentration range from 7.8 to 500μg/ml in sterile test tubes containing brain heart infusion broth (BHI) for bacteria and sabouraud dextrose broth (SD) for fungi strains. Ninety five μl of the cultures media and 5μl of the inoculum were dispensed into each well of the 96-well plates. Then 100μl from essential oil dilutions was added to the wells. A well-containing 195μl of the cultures media and 5μl of the inoculum without the test sample were used as negative control. Gentamicin and rifampin for bacteria and nystatin for fungi were also used as positive control in same conditions as described to test samples. The content of plates was mixed on a plate shaker at 300rpm for 20sec and then incubated at appropriate temperatures for 24h. Microbial growth was determined by the presence of a white pellet on the well bottom and confirmed by plating 5μl samples from clear wells on NA medium. The MIC value was defined as the lowest concentration of the plant essential oil required for inhibiting the growth of microorganisms. All tests were repeated two times (Table 2).

#### Acetylcholinesterase inhibitory assay

Acetylcholinesterase inhibitory activities of the sample were determined (14), with slight modification in a 96-well microplate. Briefly, 125μl of 3 mMDTNB [5, 5′-dithiobis (2-nitrobenzoic acid)], 25μl of 15m MATCI (acetylthiocholine iodide), 50μl of phosphate buffer (pH 8) and 25μl of the essential oil sample solution (3mg ml^−1^, in methanol) were added to 96-well plates. The absorbance was recorded at 405nm in 13sec intervals for 65 sec using a TECAN microplate reader. After that, 25μL of AChE enzyme (0.22 U ml^−1^) was added and the absorbance was measured again in 13sec intervals for 104sec. the enzyme activity was calculated from the slope of the line obtained from plotting of the absorbance against time. Any increase in the absorbance caused by non-enzymatic hydrolysis of ATCI was corrected by the recorded absorbance before addition of enzyme. Percentage of enzyme inhibition was calculated by comparing the rates for the sample to the blank (using methanol without tested sample). Physostigmine was used as the positive control (Table 3).

**Table 3.** The results of free radical scavenging, Acetylcholine esterase inhibitory and brine shrimp lethality assays of the essential oil of *Ferulago trifida*

**Samples**	**DPPH free radical scavenging assay IC_50_ (μg/ml)**	**AChE inhibitory assay IC_50_ (μg/ml)**	**Brine shrimp lethality test LD_50_ (μg/ml)**
Essential oil	111.2 ± 5.2	21.5 ± 2.2 (mg ml^−1^)	1.1 ± 0.3
BHT	21.2 ± 2.6	NA ^a^	NA
Podophyllotoxin	NA	NA	2.80 ± 0.3
Physostigmine	NA	0.8 ±0.04	NA

a Not applicable. Data are presented as the mean ± SEM of three independent experiments (P< 0.05)

#### Free radical scavenging assay

Antioxidant capacities of the essential oil of *F. trifida* were determined (15). Briefly, 2.5ml of DPPH (2, 2-diphenyl-1-picrylhydrazyl radical, Merck, Germany) solution (80μg ml^−1^ in methanol) was added to 2.5ml of sample solutions prepared in concentrations ranging from 5.0 to 9.5× 10^−3^mg/ml in methanol, and test tubes were kept in dark for 30min at 25 °C, then UV absorptions were recorded on an Optizen 2120 UV PLUS spectrophotometer at 517nm. BHT (Butylated hydroxytoluene) was used as a positive control. All tests were done in triplicate and IC_50_ values were reported as Mean± SD (Table 3).

#### Brine shrimp lethality test

General toxicity of the essential oil was evaluated by brine shrimp lethality test (BSLT) (16). For preparation of artificial seawater, 38g of sea salt was dissolved in 1.0L water and adjusted to pH 9 using sodium carbonate. The cysts of *Artemia salina* L. were hatched in sterile artificial seawater under constant aeration for 48 h at 30 °C. 50mg of essential oil were mixed with 250μl DMSO and one drop tween 80 and diluted with artificial sea-water to get 1000, 700, 500, 300, 100, 10, 2, 1, 0.5 and 0.25μg/ml concentrations in a series of tubes containing about 20 active nauplii in each. The tubes were placed in a water bath at 30 °C for 24h under light, and the surviving nauplii were then counted to obtain the concentration causing 50% lethality (LD_50_ value). Podophyllotoxin, a known cytotoxic natural compound, was applied as positive control. The test was carried out three times and LD_50_ value was reported as Mean ± SD (Table 3).

#### Cytotoxic activity

Cytotoxic activities of the essential oil of *F. trifida* was evaluated by MTT [3-(4, 5-dimethyl thiazol-2-yl)-2, 5-diphenyl tetrazolium bromide] colorimetric assay (17). Three tumor cell lines, MCF-7 (human breast adenocarcinoma), A-549 (non-small cell line carcinoma) and HT-29 (human colon adenocarcinoma) were prepared from Pasture Institute of Iran. The cell lines were cultured in Dulbecco’s Modified Eagle’s Medium (DMEM) supplemented with 10% fetal bovine serum (FBS) and 1% penicillin-streptomycin in a 5% CO_2_ incubator at 37 °C.

Cells were seeded into 96-well plates at a density of 0.5–1.5×10^4^ cells/well and incubated for 24h at 37 °C. The medium was then replaced with fresh medium containing different concentrations of essential oil and incubated for 72h at 37 °C. Then, the medium was changed by fresh medium containing MTT and incubated for 4h. During this period, MTT is reduced to formazan by living cells. Finally, the precipitated formazan crystals (purple dye) were dissolved in 200μl DMSO and determined at 570nm, in a TECAN microplate reader. Cytotoxic activity of the essential oil was defined as a 50% reduction in viability of cells (IC_50_ value). Tamoxifen was used as positive control (Table 4).

**Table 4.** The results of MTT assay of the essential oil of *Ferula gotrifida* on different cell lines

**Samples**	**Cell lines, IC_50_(μg/ml)**		

	**MCF7^a^**	**A-549^b^**	**HT-29^c^**
oil	22.0	25.0	42.55
Tamoxifen	3.6	10.7	2.50

a human breast adenocarcinoma,

b non-small cell line carcinoma,

c human colon adenocarcinoma

#### Bioassays and larval mortality

**Fig. 1.**
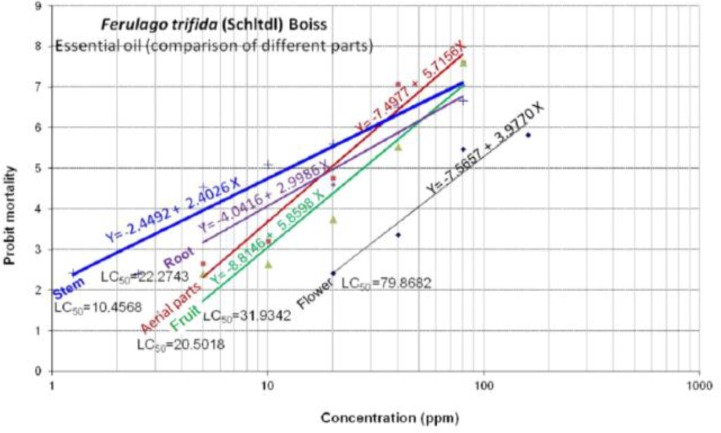
Comparison of equation, regression line and lethal concentration (LC_50_) of essential oil of different parts of *Ferulago trifida* (Apiaceae) against larvae of *Anopheles stephensi*

According to the standard method described by WHO (18), fourth instar larvae of *An. stephensi* was used for this experiment. 1ml of different concentrations (0.625, 1.25, 2.5, 5, 10, 20, 40 and 80 ppm) of essential oil (solvent: ethanol) was mixed with 224ml of water. About 25 larvae in 25ml water were added to the diluted essential oil. For control, only 1ml of ethanol with 224ml of water and 25 larvae in 25ml water were mixed and volume of the all tests and control were 250ml (19). All of the tests and control were exposed for 24h with larva. The experiment was repeated four times on different days. The percentage of mortality was reported from the average for the four replicates after 24h exposure period. From the regression line between logarithmic dose and probit mortality, the LC_50_ was determined. This investigation has been carried out in the insectarium of Department of Medical Entomology and Vector Control, School of Public Health, Tehran University of Medical Sciences, Tehran, Iran (Fig. 1).

#### Statistical analysis

Larvicidal activity was calculated from Micro Probit software (ver. 3.0). The percentages of mortality were corrected for the mortality in controls by using Abbott’s correction. From the regression line between the logarithmic dose and probity mortality, all the parameters including LC_50_ and slope values were determined (20).

In antioxidant and inhibition of acetylcholinesterase assays, One-way ANOVA and Tukey Post-hoc multi-comparison tests were used for the analyses after data normality test. Analyses were performed in triplicate and the data were expressed as mean ±SD. In cytotoxicity assay, LC_50_ (the median growth inhibitory concentration) values were calculated from the LC_50_ of dose-response curve in the sigma plot software.

## Results

### Components of the essential oil

GC and GC/MS analysis of the aerial parts essential oil were led to the identification of (86.41%) of the oil with 58 compounds. It is reach of oxygenated monoterpenes (49.30%) such as cis-verbenol, trans-verbenol (9.66%), *p*-mentha-1, 5-dien-8-ol (3.23%), isobornyl acetate (25.73%), thymol and carvacrol. E-β-caryophyllene (8.68%), Germacrene D and caryophyllene oxide are the abundant sesquiterpenes in the essential oil (Table 1).

### Antimicrobial activities

The results of essential oil effects on eleven bacteria and fungi were reported as Minimum Inhibitory Concentration (MIC) and Inhibition zone in diameter (mm) (Table 2). Rifampin, gentamycin, and nystatin were used as positive control against bacterial and fungal strains, respectively. The results of positive controls (rifampin, gentamycin, and nystatin) and negative control (DMSO) also are shown in Table 2.

### Antioxidant, AChE inhibitory and Brine shrimp lethality

In free radical scavenging assay with DPPH method, the volatile oil (IC_50=_ 111.5.2μg/ml) showed no antioxidant activity in comparison to the BHA (IC_50_=21.2μg/ml).

AChE inhibitory of the oil (22.5mg ml^−1^) versus physostigmine as standard (0.8μg ml^−1^) was very low. General toxicity of the oil was evaluated by brine shrimp lethality test, and the sample with LD_50_= 1.1μg/ml demonstrated strong toxic effect in comparison to podophyllotoxin with LD_50_= 2.8μg/ml (Table 3).

### Cytotoxic effect

The effects of *F. trifida* essential oil on the proliferative response of the MCF7 (human breast adenocarcinoma), A-549 (non-small cell line carcinoma) and HT-29 (human colon adenocarcinoma) cell lines have been analyzed by treating the cells with different concentrations of the volatile oil and significant decrease in cell lines proliferation in comparison with tamoxifen as positive control were observed in Table 4.

### Larval mortality

The larvicidal activity of the essential oils from aerial parts, flower, fruit, leaf, stem, and root of the *F. trifida* against *An. stephensi* larvae were examined under laboratory conditions and the results are presented in Fig. 1. Stem oil of the *F. trifida* was the most effective one against *An. stephensi* with LC_50_= 6.51ppm.

## Discussion

The aerial part of *F. trifida* was collected around Ovan Lake located in Alamut region, Qazvin province, Iran in July 2014. Hydro-distillation of the air-dried of aerial parts of *F. trifida,* yielded 1.5% (v/w) of the oil. Analysis of the volatile oil by GC and GC/MS resulted in fifty-eight compounds, representing 86.41% of the total oil. The result showed the essential oil of the aerial part was rich in monoterpenes (55.75%) that oxygenated monoterpenes (49.30%) were dominant. The main components were isobornyl acetate (25.73%) and E-verbenol (9.66%) but amount of the Z-beta ocimene was 3.33% whereas it is the abundant monoterpene in the oil of some *Ferulago* species (21–24, 6). In *F. aucheri*, *F. mughlae* and *F. sandrasica* essential oils α-pinene were the major compound and in essential oil of *F. macroseiadia*, *F. sylvatica* and *F. bernardii*, methyl carvacrol, *p*-cymene and 2, 4, 5-trimethyl benzaldehyde were reported as important components (22, 7). In our study, amounts of the α-pinene were only (0.42%), and there were not methyl carvacrol, *p*-cymene and 2, 4, 5-trimethyl benzaldehyde in the essential oil. In *F. trifida* there was 20.41% sesquiterpenes, which beta-caryophyllene (8.68%), Germacrene D (2.18%) and caryophyllene oxide (2.47%) were abundant.

*Ferulago trifida* aerial part volatile oil showed strong antimicrobial activity with MIC near gentamycin against all bacterial strains except *E.coli*, and antifungal activity on *A. brasiliensis* with MIC equal nystatin. *A. niger* and *C. albicans* had shown resistance to the oil. The essential oil of *Ferulago* genus showed different results depending on the compounds contained in oil (7, 21, 25, 26).

In our study, on the AchE inhibitory properties, *F. trifida* oil did not show significant effect versus physostigmine as standard. We did not observe any report about AchE inhibitory activity from essential oil of *Ferulago* genus.

The antioxidant assessment of oil of the *F. trifida* has demonstrated it has low level free radical scavenging activity because it’s IC_50_ (111.2μg/ml) was less than BHT (IC_50_= 21.2μg/ml) as standard. Such investigation has been done on some species of this genus (27).

The brine shrimp lethality test is considered as an inexpensive, simple, rapid and effective method for preliminary assessment of toxicity and as a guide for the detection of cytotoxic, anti-tumor and pesticidal compounds (28). Table 3 demonstrates the result of genera50 of l toxicity of the sample. Aerial part essential oil of the *F. trifida* with LD_50_= 1.1 μg/ml showed more toxicity against podophyllotoxin (IC_50_= 2.8μg/ml) as standard. The main sesquiterpenes of the essential oils such as β-caryophyllene, germacrene D, caryophyllene oxide etc. may be involved in the toxic effects of the tested essential oil (29).

In this investigation, the MCF7 human breast adenocarcinoma, A-549 non-small cell line carcinoma and HT-29 human colon adenocarcinoma cell lines were treated with different concentrations of *F. trifida* aerial parts essential oil and the cell viability were measured for 24 and 72h as described in the experimental part. The results of these measurements are shown in Table 4. Essential oil of *F. trifida* showed interesting toxicity with IC50≥40μg/mlon cancerous cell lines, although it was not similar to tamoxifen activity.

Other review on the literature for the cytotoxic and anticancer effects of various species *of Ferulago* essential oil, (30) has demonstrated that the essential oil of *F. carduchorum* showed potential cytotoxic selectivity on T47D cell line similar to methotrexate (positive control). The present study showed that the essential oils obtained from *F. tifida* different parts e.g. stem, root, aerial part, fruit and flower could induce 50% mortality in the larvae of *An. stephensi* at a very low concentrations (10.46, 22.27, 20.50, 31.93 and 79.87ppm) respectively. The essential oils of some plants such as *Cymbopogon nardus*, *C. flexuosus*, *C. martini*, *Lavandula officinalis*, *Menthaarvensis*, *Racinus communis*, *Eucalytus globules*, *Eugenia caryophyllus*, *Ocimum basilicum*, *Melia azardirachta*, *Cannabis sativa* demonstrated LC_50_ values of 105.4, 91.4, 100.0, 83.6, 83.8, 113.0, 98.5, 96.5, 80.0, 88.5, and 27.0ppm respectively, against the larvae of the *An. stephensi* (31–34). There are several studies on larvicidal activities of different plants against malaria vectors in Iran (35–59, 30). Therefore, the *F. tifida* aerial parts essential oil has strong activity against *An. stephensi*.

## Conclusion

The essential oil of *F. trifida* aerial parts, collected from Qazvin Province of Iran, has demonstrated some biological activities including antibacterial effects on Gram positive, Gram-negative and 1 fungi, high general toxicity on the brine shrimp lethality test, cytotoxic effects on three cancerous cell lines, and has remarkable larvicidal properties on fourth instar larvae of *An. stephensi*. Therefore, it is worthwhile to study on the larvicidal properties of its essential oil by isolating and identifying the active components that cause larval mortality, and their field trials.
